# Pain-Related Behavior and Pain Perception Associated with Intraosseous Local Anesthesia (QuickSleeper 5^®^) in Pediatric Patients: A Randomized Controlled Clinical Trial

**DOI:** 10.3390/children12010065

**Published:** 2025-01-07

**Authors:** Zeyad A. AlRaddadi, Latifa A. AlHowaish, Ayman M. Sulimany

**Affiliations:** Department of Pediatric Dentistry and Orthodontics, College of Dentistry, King Saud University, Riyadh 11255, Saudi Arabia; lalhowaish@ksu.edu.sa (L.A.A.); asulimany@ksu.edu.sa (A.M.S.)

**Keywords:** pain-related behavior, pain perception, local anesthesia, Quicksleeper 5, pediatrics

## Abstract

Background: Managing pain during dental procedures is crucial, particularly for children, as pain can induce anxiety. Local anesthesia is the most anxiety-inducing procedure in pediatric patients. Consequently, this study aimed to assess and compare the pain-related behaviors and perceptions associated with two anesthesia techniques for children: traditional local anesthesia and intraosseous local anesthesia administered via the QuickSleeper 5^®^ system. Methods: A split-mouth randomized clinical trial was conducted involving 33 children aged 4–9 years. Each participant received both traditional local anesthesia and intraosseous local anesthesia with QuickSleeper 5 across two visits. Pain-related behaviors were evaluated by calibrated pediatric dentists through video recordings, and pain perceptions were assessed using the Wong–Baker Faces Pain Rating Scale. Heart rate measurements offered objective insights into patients’ anxiety; finally, the time needed to administer anesthesia was recorded. Results: This study found no statistically significant differences between traditional local anesthesia and intraosseous local anesthesia with QuickSleeper 5 regarding pain perception, heart rate, or pain-related behaviors, indicating that both techniques are effective at minimizing discomfort. However, QuickSleeper 5 demonstrated a significantly shorter administration time, enhancing the procedural efficiency of pediatric dentistry. Conclusions: The QuickSleeper 5 system is a valuable tool for pediatric dental care, delivering comparable comfort levels to traditional anesthesia while significantly reducing the time that is required for administration. The QuickSleeper 5 system’s efficiency advantage could make it the preferred choice for treating children, especially given the stress that is often associated with local anesthesia and the need for quick, smooth procedures in pediatric care.

## 1. Introduction

Pain control is critical during dental treatment, especially when treating pediatric patients. Maintaining control of an excruciatingly painful condition is essential, and minimizing discomfort during each visit is a vital requirement [1]. Local anesthesia (LA) is the primary technique utilized in dentistry to mitigate the discomfort and pain associated with dental procedures. A significant number of individuals delay dental treatment because of their apprehension regarding local anesthesia. Children exhibit the most pronounced reactions to injections during dental procedures [1]. A study conducted in Saudi Arabia found that more than 50% of the studied subjects suffer or may suffer from dental phobia, while the fear of pain and dental injections were the most common causes reported for this fear [2]. Dental fear and anxiety might lessen individuals’ likelihood of utilizing oral health services, thus hindering the early detection and treatment of dental conditions. Aggregated fear and anxiety about dental visits in children often lead to negative oral health outcomes, because these children are more susceptible to the consequences of untreated dental conditions [3].

Hence, selecting the least painful LA administration technique is crucial [4]. Furthermore, the characteristics of the anesthesia and the needle that is used during injections may influence the patient’s pain levels [5,6]. In addition, reduced anesthetic delivery rates have also been shown to reduce the pain associated with injections [7].

The computer-controlled local anesthetic delivery (CCLAD) system facilitates the gradual infusion of a local anesthetic solution, resulting in reduced pain during administration. A randomized clinical study evaluated the pain perception associated with the use of CCLAD, which was shown to induce significantly less discomfort than a standard anesthetic syringe [8]. The dental literature indicates that the utilization of CCLAD diminishes pain perception, mitigates disruptive pain behavior, and lessens the necessity for restraint in children undergoing local dental anesthesia [9,10].

The traditional buccal infiltration technique (BIT) is usually sufficient to anesthetize mandibular primary molars in children up to five years of age [11]. With older children, the success rate of mandibular BIT in mandibular molars decreases [12]. Several approaches exist to alleviate pain during an infiltration procedure: topical analgesic gels, distraction techniques, warming anesthetic gels, buffering the anesthetic agent, and reducing the injection speed. The effectiveness of these treatments in alleviating pain remains debatable [9,13].

The intraosseous technique (IOT) is administered into porous alveolar bone [14]. The American Academy of Pediatric Dentistry (AAPD) asserts that the IOT is a safe procedure for pediatric patients [5,6]. The IOT offers multiple advantages, such as reduced local anesthetic volume, a rapid onset of action (30 s), and a decrease in postoperative complications [14]. When combined with a computer-controlled device that makes osseus penetration and anesthetic distribution easier, this method can be used instead of or in addition to BITs in young children and adolescents [15]. A review article established that computer-assisted intraosseous anesthesia serves as an effective primary method for administering anesthesia to one or two mandibular posterior teeth. The combination of 0.6–0.8 mL of 4% articaine with 1:200,000 epinephrine exhibits a high success rate, ease of administration, and considerable patient comfort [16].

Developments in CCLAD have resulted in the creation of more user-friendly products for both the patient (by lowering pain and fear) and the dentist. The Wand has been available on the market for the longest duration, followed by the introduction of various CCLAD systems such as QuickSleeper, Comfort Control Syringe, and Meg-inject [17].

The QuickSleeper 5^®^ (QS5; Dental Hi Tec, Cholet, France) is a CCLAD system comprising a handpiece designed with a pen grip for enhanced accuracy, a control unit, and a foot pedal. The device is intended for local injections at a consistent rate and pressure. A single rotation is typically sufficient to insert the needle into the cancellous bone, necessitating no physical exertion from the operator. The device enables the operator to perforate the bone and inject the anesthetic solution while minimizing discomfort, trauma, and tissue heating. The anesthetic solution is administered incrementally, drop by drop. The device utilizes specially designed needles that facilitate both perforations and injections using a single needle [15,16,17,18].

This study aims to evaluate and compare pain-related behavior and pain perception during local anesthesia injections in the BIT and IOT utilizing 4% articaine (Septodont, Paris, France) with QS5 (Dental Hi Tec, Cholet, France).

## 2. Materials and Methods

### 2.1. Ethical Approval

This study was approved and registered with the College of Dentistry Research Center (No. PR 0140). Additionally, the study was registered with the Saudi Food and Drug Authority under application number 2226. Finally, the study’s protocol was registered in BioMed Central (BMC) under the number ISRCTN14600773.

### 2.2. Study Design

**Figure 1:**
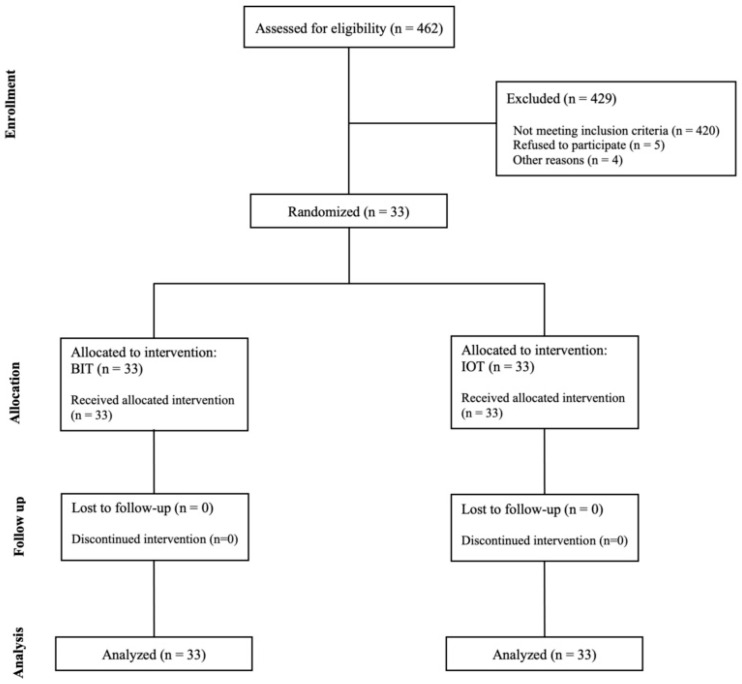
Consort diagram of the sample selection for the two local anesthetic techniques, a traditional technique (BIT) and intraosseous, given via QuickSleeper 5 (IOT).

This split-mouth randomized clinical trial follows the CONSORT guidelines (Figure 1) [19]. The study was conducted at the Dental University Hospital (DUH) at King Saud University (KSU), Riyadh, Saudi Arabia.

### 2.3. Sample Size Calculation

The sample size was calculated using SPSS Version 26.0 (IBM Inc., Chicago, IL, USA). Using the pain perception associated with LA as the main outcome for the calculation, there should be at least 33 patients to detect an effect size of 0.6 with 0.90 power and an alpha = 0.05.

### 2.4. Sample Selection

The sample was selected from children who attended pediatric dental clinics at DUH with definitely positive or positive behavior according to the Frankl behavior scale [20] and who had two mandibular primary molars that were indicated for pulpotomies and stainless steel crowns (SSCs), without any signs or symptoms of pulpal degeneration. The study’s protocol, risks, and benefits were explained to the parents or legal guardians, and informed consent was obtained from them if they agreed to participate.

### 2.5. Inclusion Criteria

- Aged 4–9 years.
- Physically and mentally healthy.
- Positive or definitely positive behavior; this was assessed by taking the history of the patient. If the patient had no previous dental treatment, a treatment was provided to the patient to assess that individual’s behavior.
- No contraindication for the use of local anesthesia.
- Not taking any analgesic before the appointment for at least 24 h [21,22].
- No infection or inflammation on either side of the lower arch and two lower primary molars, indicated for pulpotomies and SSCs.
- No history of spontaneous or persistent pain.
- No pathological mobility or tenderness to percussion.
- No swelling or sinus tract.
- No widening of the periodontal ligament space.
- Restorable teeth.

### 2.6. Randomization

Because of the nature of the split-mouth design, patients served as their own controls. Each tooth fulfilling the inclusion criteria regarding the mandibular arch was randomly assigned to one of two groups, the control group (traditional syringe) or the experimental group (intraosseous given by QuickSleeper5), using a list of random numbers that were generated online with a randomization program (http://www.randomizer.org). During the first appointment, the patient was given one type of the mentioned LA techniques (BIT or IOT), depending on the randomization; during the second visit, the patient was given the other technique. The time period between the two visits was a minimum of 7 days and a maximum of 30 days. This time period was given to manage the patients’ compliance. If the patient did not arrive within this time limit, that individual was excluded from the trial. The time period was registered for each patient. Any patient was excluded from this study if that individual became uncooperative during either visit or underwent dental treatment between the two visits. Due to the nature of the study, only the statistician could be blinded in the trial. The operator, evaluators, and patients could not be blinded to the anesthetic techniques used.

### 2.7. Clinical Procedure

#### 2.7.1. Screening Visit

The patients were selected from the list of patients who arrived for screening at the Department of Pediatric Dentistry at DUH. The primary investigator screened the patients who were booked for regular screening. A thorough medical and dental history was taken for all patients, followed by a full-mouth examination, including clinical and radiographic assessments. Standardized periapical and bitewing radiographs were taken for the teeth indicated for pulpotomy using a Snap A-Ray device (Dentsply Sirona, Johnson City, TN, USA) to rule out any inflammation or infection related to the lower primary molars. Patients who fulfilled the inclusion criteria were booked for the intervention procedure following a detailed explanation of the study and after informed consent from the legal guardian was obtained. Patients who did not meet the inclusion criteria or who did not agree to participate were booked for treatment in the department.

#### 2.7.2. Informed Consent

Before starting the procedure, the operator doublechecked and reviewed the informed consent with the legal guardian and gave them a chance to ask questions.

#### 2.7.3. Behavioral Management Technique

Only basic non-pharmacological behavioral management techniques were used (tell–show–do, positive reinforcement, nonverbal communication, communication and communicative guidance, and ask–tell–ask) with the patient during the study. No advanced or pharmacological techniques were employed. In case the patient did not cooperate, a referral was made for the patient to receive pharmacological behavioral management techniques (sedation or general anesthesia) in accordance with the patient’s treatment needs.

### 2.8. Local Anesthesia Technique

#### 2.8.1. Procedure for the Traditional Infiltration Technique

A single operator performed all injections. For the BIT, the topical anesthetic gel Benzocaine 20% (Prime Dental, Chicago, IL, USA) was applied for 30 s after drying the mucosa with gauze before administering the injection [23]. A 21 mm long, 30-gauge needle was used. The needle’s tip was placed in the buccal vestibule and then directed to the apex of the tooth, after which the lingual gingiva was anesthetized via intrapapillary injection. A total of 1 mL Septanest SP of 4% articaine hydrochloride with 1:100,000 adrenaline (Septodont, France) was used.

#### 2.8.2. Procedure for Intraosseous Injection

All IOT injections were performed by the same operator who conducted the BIT. The QS 5 system was used to preform IOT following the three-step procedure described by the manufacturer. No topical anesthesia was given. The device was introduced to the child as a “magical pen”. First, the mucosa was anesthetized by inserting the DHT Effitec Needle (Dental Hi Tec, Cholet, France) at a 15–20° angle to the buccal mucosa, with the flat part of the bevel facing the mucosal surface approximately 1–3 mm below the distal septum and adjacent to the tooth to be anesthetized. If the distal could not feasibly be injected, the needle was inserted into the mesial side. A few drops of local anesthetic solution were injected to induce superficial anesthesia, allowing the needle to be inserted at a 90° angle. The needle then rotated until the cancellous bone was penetrated. Next, a 1 mL anesthetic solution, 1 mL/min Septanest SP of 4% articaine hydrochloride with 1:100,000 adrenaline (Septodont, France), was injected. A 9 mm long, 30-gauge needle was used.

#### 2.8.3. Vital Sign Monitoring

To establish the baseline for each patient, heart rate (HR) readings were taken at the beginning of each visit using a Mindray VS-600 monitor (Shenzhen, China). In addition, the HR was recorded during the administration of local anesthesia, because it is associated with anxiety. This provides an objective finding regarding the patient’s feelings during the administration of LA to rule out bias from the evaluators, in addition to ruling out bias in the patient’s subjective self-reporting of pain.

#### 2.8.4. Pain-Related Behavior

Before the start of the data collection phase, two pediatric dentists working in the Department of Pediatric Dentistry were calibrated to use the index for evaluating pain-related behavior. The procedures were video-recorded, and two calibrated pediatric dentists evaluated the behavior of the patient using five pain-related behaviors during the injection. The videos were edited with a sound indicator to ensure that both evaluators checked the patient’s behavior simultaneously. These behaviors were recorded as present or absent at every 15 s interval of the entire injection period [24]:

1. Bodily movement, defined as the movement of an extremity greater than 15 cm or the turning of the body.
2. Muscle tension, defined as visible tension in the hands (white knuckles) or tension throughout the body.
3. Crying or screaming.
4. Verbal protest.
5. Bodily resistance, when necessary, to maintain control of the child.

To calculate the mean score for pain-related behaviors, the occurrences of all five behaviors were added and divided by the number of intervals. Intraexaminer and interexaminer reliabilities were assessed using Cohen’s kappa.

#### 2.8.5. Pain Perception

The Wong–Baker Faces Pain Rating Scale [25] was used to evaluate the children’s perception of pain following the injections. This index has been validated for the Saudi population [26]. This rating scale is appropriate for children aged three and above. Before administering anesthesia, the operator explained the faces in the scale to the patient with scripted instructions that were read to all patients, accompanied by pointing to each face and describing the associated pain intensity with the corresponding words. Immediately following the administration of local anesthesia, the child was asked, “Which looks like how you felt during the injection?” to select the face that best described their pain. The corresponding number was recorded.

### 2.9. Statistical Analysis

The data were analyzed using SPSS Version 26.0 (IBM Inc., Chicago, IL, USA). Descriptive statistics, including mean, standard deviation, frequencies, percentages, and range, were employed to characterize the quantitative and categorical variables. Paired sample *t*-tests were employed to compare quantitative outcome variables against categorical study variables, categorized into two groups. Kappa statistics were utilized to assess intra- and interobserver agreements. A *p*-value of ≤0.05 was utilized to indicate the statistical significance of the results.

## 3. Results

The pain perceptions and pain-related behaviors of the 33 subjects were assessed using two anesthesia techniques (BIT and IOT); Table 1 illustrates the descriptive statistics. The average age of the 33 study participants was 6.36 years, with 60.6% being between 4 and 6 years, and most being female. The research was conducted during two visits with the study participants. More than half of the sample (57.6%) had undergone previous dental treatment. In the trial, 28 (44.4%) first primary molars were treated, while 38 (56.6%) second primary molars were treated, more details are presented in the table. The interval between two visits was 7 days or fewer for 18 participants, whereas 15 subjects had intervals of 8 to 15 days and greater than 15 days, with a mean of 11.33 days (6.55) (see Table 1).

Table 2 presents a comparison of the effects of the two anesthesia techniques (BIT and IOT) on several outcome variables, including heart rate difference, pain perception, pain-related behavior, and the duration of anesthesia administration. The BIT produced a mean heart rate difference of 16.97 (±10.40), whereas the IOT exhibited a marginally higher mean of 20.82 (±18.11). A *p*-value of 0.294 indicated that the difference between the two techniques was not statistically significant. In terms of pain perception, patients who underwent the BIT exhibited a mean score of WBS 1.55 (±0.31), whereas those utilizing the IOT recorded a mean score of 1.82 (±1.76). The difference did not reach statistical significance (*p* = 0.517). The mean score for the BIT regarding pain-related behavior was 0.25 (±0.31), whereas the IOT exhibited a marginally higher score of 0.42 (±0.51). However, this difference was not statistically significant (*p* = 0.104). A notable difference was observed in the duration of anesthesia administration. The BIT required an average of 160.39 (±32.38) seconds, while the IOT demonstrated greater efficiency, averaging 132.88 (±46.31) seconds. The *p*-value of 0.007 demonstrates that the observed difference in speed was statistically significant, indicating that the IOT is considerably faster.

Table 3 demonstrates the mean values of the five components of pain-related behavior (movement, muscle tension, crying, verbal protest, and resistance). For the muscle variable, the BIT showed a higher mean value (1.0 ± 0.94) compared to the IOT (0.76 ± 0.86). Although this difference seemed apparent, the *p*-value (0.283) indicated that it was not statistically significant. Similarly, for the crying variable, the IOT had a slightly higher mean (0.47 ± 0.98) than the BIT (0.35 ± 0.69), but this difference was also not significant (*p* = 0. 567). The mean overall behavior was 0.344 ± 0.46 and 0.324 ± 0.42 for the BIT and IOT, respectively, with no statistically significant difference (*p* = 0.8542).

Two independent observers evaluated the pain-related behavior and its components. A statistically significant strong agreement existed between the two observers (kappa = 0.95; *p* < 0.0001) concerning their evaluation of pain-related behavior and its five components. The intraobserver agreement was strong for both observers, as indicated by kappa values greater than 0.90. A *p*-value of ≤0.05 was used to denote the statistical significance of the results.

## 4. Discussion

This split-mouth randomized controlled clinical trial was intended to assess pain-related behavior and pain perception in pediatric patients during the administration of the IOT using the QuickSleeper 5^®^ system compared to the BIT approach. Limited studies have compared intraosseous anesthesia in the pediatric population. Therefore, a study assessing the patients’ pain-related behaviors during this anesthetic technique was necessary. Furthermore, no study has compared the IOT using QuickSleeper 5 to buccal infiltration in pediatric patients. No research has standardized the injection site or the method of administering local anesthesia. The aim was to evaluate and compare the two techniques with regard to pain-related behavior and pain perceptions by assessing the pain levels subjectively and objectively.

In this trial, we choose patients who needed standard treatments, mainly pulpotomy and stainless steel crowns. Any patient who needed more invasive procedures such as tooth extraction or surgical treatments was not included in the study, as invasive procedures might influence the fear and anxiety of the patient.

To limit inter-individual variability and enable a balanced comparison between the two anesthetic techniques, a split-mouth design was implemented. Each patient acted as their own control. A total of 33 healthy and cooperative pediatric patients aged four to nine years were recruited, with each receiving both forms of anesthesia across two visits. Pain-related behaviors were assessed using a calibrated observational index, while the children’s subjective pain perception was evaluated using the Wong–Baker Faces Pain Rating Scale. Moreover, heart rate was assessed to offer an objective evaluation of the patients’ responses to fear and pain throughout the administration of anesthesia. Lastly, the time at which anesthesia was given was recorded, accounting for age, gender, previous dental experience, and their effect on behavior.

Age, gender and previous experience did not affect the behavior of the patient and were statistically insignificant. The main finding of this trial was that the pain perception and pain-related behavior associated with the IOT given with the QuickSleeper 5^®^ system and the BIT were equivalent. No statistically significant differences were detected between the two techniques for these measures. This indicates that both methods are comparably successful at reducing discomfort in pediatric patients during dental treatment. These findings coincide with previous studies that evaluated the behavior and cooperation between various CCLAD systems in the pediatric population. Two trials were conducted evaluating the Wand vs. conventional techniques in children who experienced no difference in pain perception or anxiety in the conventional or Wand group [27,28]. In addition, one study evaluated the physiological parameters in 6–9-year-old patients. There were no differences between the Wand and traditional techniques [13]. Gibson et al. published the findings of a study involving 62 children ranging in age from five to thirteen years who required local dental anesthesia. Patients received either a syringe injection or CCLAD at random. The authors concluded that injecting children with the CCLAD instrument reduced their disruptive behavior during injections [11]. However, this study did not standardize the amount of local anesthesia or the injection site. Furthermore, the trial lacked clear criteria to assess the patients’ behavior.

Two studies evaluated the Morpheus local anesthesia system (Meibach Tech, Sao Paulo, Brazil) and found that it did not affect the levels of pain, disruptive behavior, anxiety, and physiological parameters in children compared to traditional techniques [29,30]. These studies used a parallel design to evaluate the behavioral differences among pediatric patients, without indicating the technique for which the Morpheus system was used. In addition, none of these studies used the same injection site across the groups.

Other studies have evaluated other CCLAD systems and found less disruptive patient behavior. A 2006 study assessed patients’ reactions while receiving LA in the anterior area using the Wand system compared to the traditional method. Although they found that the Wand group exhibited better behavior during the injection, all patients were sedated with hydroxyzine and nitrous oxide [31]. From our perspective, the sedative agents may have compromised the findings of this study by masking the patients’ behavior. A crossover study examined the differences between traditional local anesthesia and the Wand system in pediatric dentistry by examining pain perception and heart rate responses. The Wand system reduced pain, anxiety, and heart rate increases in children between the ages of seven and fifteen [7]. A limitation of this study is the recruitment of older age group; thus, their findings might not be generalizable to younger age groups.

Regarding pain perception, a 2020 study evaluated the Morpheus system in young patients aged 5–8 years; the authors found no statistically significant differences [29]. The same research group conducted a 2021 study on an older sample aged 9–12 years and found similar results [30]. Neither study mentioned the site of the injection technique (i.e., infiltration or nerve block). Another trial found that pain perception was not statistically significant in 8–12-year-olds when they compared the Wand to the traditional technique while administering buccal infiltration [28]. This finding for the Wand system aligns with a study that assessed the pain perceptions of children aged six, nine, and twelve and found no significant differences between it and the traditional syringe [13].

Very few studies have compared pain-related behaviors between buccal infiltration and the IOT given to children by the QS 5 system. One study evaluated pain while using the IOT with the QS 5 system and found minimal discomfort; however, this study had no control group for comparison [15]. A study employing the QS 5 system and traditional methods found that the QS 5 group experienced less pain [18]. However, this study’s population differed from our age group. In our study, the oldest participant was nine years old, whereas in the aforementioned study, the age range extended up to fifteen years. In addition, they compared regional anesthesia, not the infiltration technique, versus the IOT. Lastly, they did not consider the child’s behavior while recruiting.

The duration of the anesthesia procedure was significantly shorter with the IOT than the BIT. This finding aligns with prior research emphasizing this feature in CCLAD systems, which use a slow, controlled flow for injections [7,10]. By reducing the time that is needed for local anesthesia, the quick application of IOT systems may provide noticeable advantages in pediatric dentistry settings by lessening appointment times, hence enhancing the dental experience for patients. However, the extended duration that was needed to finish administering the BIT injection did not demonstrate any significant negative influence on the children’s behavior in this sample.

Another key observation was the high level of agreement between the two independent observers in evaluating pain-related behaviors, as depicted by the strong intra- and interexaminer reliability (kappa > 0.90). This reinforces the validity and reliability of the observational pain behavior index used in this study, ensuring confidence in the accuracy of our findings.

For most patients, QuickSleeper 5 required only one rotation to penetrate the bone. However, the QuickSleeper 5 system has several limitations. The system has a learning curve; this is why the operator had to be given a lecture on how to use it with patients before proceeding with the study sample. Moreover, the distal side of the primary second molar may not always be suitable for the injection, because the permanent first molar is erupting, and insufficient bone is available for administering the IOT. Moreover, bone may obstruct the needle, necessitating replacement, which was encountered in this trial. The needle needed to be changed, because local solutions were not passing through the needle due to bone obstructions. One of the patients experienced two instances of bone blocking the needle. Upon reviewing the patient’s radiograph, we found that the crestal bone highly condensed, showing an increase in radiopacity, which could explain the blockage.

Our study’s design has a few limitations. For example, blinding was impossible for the operator, patients, or evaluators. Although some studies chose to cover patients’ eyes while administering local anesthesia, we opted not to do so due to the risk of triggering the patients’ anxiety. A multicenter study would provide more robust evidence than a single-centered one.

A recently published study found a wide range of other variables that could possibly affect a child’s behavior during dental treatment, such as the body mass index [32]. Therefore, we encourage including such variables in future studies.

Conversely, some strengths must be noted in our design, such as single-trained operators performing all the procedures, calibrated evaluators with excellent kappa agreement, and a split-mouth design to reduce inter-individual variabilities. Moreover, the analysis accounted for the patients’ previous experience. The same amount of local anesthesia was given to patients. In addition, pain was evaluated using three methods: a calibrated evaluator, patient response, and physiological parameters.

## 5. Conclusions

This study adds valuable insight to the existing dental literature on CCLAD systems, highlighting the QuickSleeper 5^®^ as an efficient tool for pediatric dentistry, despite pain perception, heart rate, and pain-related behaviors that were comparable to those for traditional methods. The speed advantage could make it the preferred choice for treating children, especially given the stress that is often associated with local anesthesia and the need for quick, smooth procedures in pediatric care.

## Figures and Tables

**Table 1:** Descriptive statistics of demographic characteristics, tooth treated in each visit, and time period between the visits of study subjects and the previous treatment (n = 33).

Study Variable	Number, n (%)
Age (in years), mean ± SD	6.36 ± 1.41
Time between visits (in days), mean ± SD	11.33 ± 6.55
Gender	
Male	13 (39.4)
Female	20 (60.6)
Previous treatment	
Yes	19 (57.6)
No	14 (42.4)
Tooth number during the first visit	
First primary molar	13 (39.4)
Second primary molar	20 (60.6)
Tooth number during the second visit	
First primary molar	15 (45.5)
Second primary molar	18 (54.5)
Time between visits (in days)	
≤7	18 (54.5)
8–15	8 (24.2)
>15	7 (21.2)

**Table 2:** Comparison of proportions and mean values of outcome variables between two types of anesthesia techniques and tooth number.

Study Variables	Outcome Variables, Mean ± SD			
	Heart Rate Difference (Min and Max)	Pain Perception	Pain-Related Behavior	Time for Anesthesia (in Seconds)
Type of anesthesia technique				
BIT	16.97 ± 10.40	1.55 ± 0.31	0.25 ± 0.31	160.39 ± 32.38
IOT	20.82 ± 18.11	1.82 ± 1.76	0.42 ± 0.51	132.88 ± 46.31
*p*-value	0.294	0.517	0.104	0.007 *
Tooth number				
First primary molar	21.82 ± 16.16	1.43 ± 1.55	0.34 ± 0.47	137.86 ± 34.16
Second primary molar	16.73 ± 13.50	1.87 ± 1.79	0.33 ± 0.40	153.11 ± 46.33
*p*-value	0.169	0.300	0.898	0.146

* Statistically significant. BIT: traditional buccal infiltration technique. IOT: intraosseous technique.

**Table 3:** Comparison of the mean values of each component of pain-related behavior between the two types of anesthesia techniques.

Study Variables	Type of Anesthesia Technique, Mean ± SD		*p*-Value
	BIT	IOT	
Move	0.35 ± 0.63	0.36 ± 0.58	0.945
Muscle	1.0 ± 0.94	0.76 ± 0.86	0.283
Crying	0.35 ± 0.69	0.47 ± 0.98	0.567
Verbal	0.02 ± 0.19	0.03 ± 0.34	0.883
Resist	0.0	0.0	–
Mean of overall behavior	0.344 ± 0.46	0.324 ± 0.42	0.8542

BIT: traditional buccal infiltration technique. IOT: intraosseous technique.

## Data Availability

The raw data supporting the conclusions of this article will be made available by the authors on request. The data are not publicly available, as the data may be subject to restrictions, including compliance with ethical guidelines or privacy concerns. Requests will be evaluated on a case-by-case basis to ensure appropriate use and adherence to any applicable regulations.
